# Supplementary material for: Detrimental impact of sulfide on the seagrass Zostera marina in dark hypoxia
Source: PLoS One. 2023 Dec 7;18(12):e0295450. doi: 10.1371/journal.pone.0295450 (PMC10703230; doi:10.1371/journal.pone.0295450)
Supplement: S1 File — (DOCX) [file pone.0295450.s002.docx]

**Supporting Information**

**Detrimental impact of sulfide on the seagrass Zostera marina in dark hypoxia**

Harald Hasler-Sheetal* ^1,2^

^1^Nordcee, University of Southern Denmark, Denmark.
^2^VILLUM Center for Bioanalytical Sciences, University of Southern Denmark, Denmark.

* corresponding author: hasler@sdu.dk
Nordcee, University of Southern Denmark, Campusvej 50, 5230 Odense M, Denmark

**Contents:**

Table S1: Results of 2-way ANOVA testing the effects of sulfide exposure and time on metabolite levels.

Table S2: Results of 2-way ANOVA testing the effects of sulfide exposure and time on Phytol and MDA level.

| Table S1: Results of 2-way ANOVA testing the effects of sulfide exposure and time on metabolite levels; Test statistics underlying Fig 3. | | | | | | | | | |
| --- | --- | --- | --- | --- | --- | --- | --- | --- | --- |
| Source of variation |  | Treatment | |  | Time | |  | Treatment x Time | |
|  |  | F | p |  | F | p |  | F | p |
| Alanine |  | 7246.2 | >0.001 |  | 905.21 | >0.001 |  | 274 | >0.001 |
| Cysteine |  | 4948.7 | >0.001 |  | 28.796 | >0.001 |  | 274.8 | >0.001 |
| Glutathione |  | 2061.7 | >0.001 |  | 17.133 | >0.001 |  | 97.02 | >0.001 |
| Lactate |  | 1934 | >0.001 |  | 1230.5 | >0.001 |  | 73.91 | >0.001 |
| Serine |  | 1794.8 | >0.001 |  | 536.83 | >0.001 |  | 156.8 | >0.001 |
| 2-Oxoglutarate |  | 1759.9 | >0.001 |  | 686.5 | >0.001 |  | 147.0 | >0.001 |
| Proline |  | 1744.1 | >0.001 |  | 77.029 | >0.001 |  | 113.6 | >0.001 |
| GABA |  | 1652.3 | >0.001 |  | 423.03 | >0.001 |  | 376.9 | >0.001 |
| Succinate |  | 1097.3 | >0.001 |  | 1131.8 | >0.001 |  | 251.9 | >0.001 |
| Valine |  | 1015.9 | >0.001 |  | 67.6 | >0.001 |  | 137.7 | >0.001 |
| Fumarate |  | 960.64 | >0.001 |  | 70.195 | >0.001 |  | 101.9 | >0.001 |
| Tyrosine |  | 781.27 | >0.001 |  | 208.9 | >0.001 |  | 101.7 | >0.001 |
| Aspartate |  | 751.96 | >0.001 |  | 388.57 | >0.001 |  | 50.8 | >0.001 |
| Pyruvate |  | 601.33 | >0.001 |  | 1066.4 | >0.001 |  | 57.79 | >0.001 |
| Glucose |  | 542.21 | >0.001 |  | 137.56 | >0.001 |  | 12.04 | >0.001 |
| Malate |  | 514.42 | >0.001 |  | 66.886 | >0.001 |  | 102.2 | >0.001 |
| Glycine |  | 453.36 | >0.001 |  | 208.42 | >0.001 |  | 25.01 | >0.001 |
| Fructose |  | 450.12 | >0.001 |  | 803.62 | >0.001 |  | 19.53 | >0.001 |
| Glutamate |  | 157.37 | >0.001 |  | 64.227 | >0.001 |  | 11.75 | >0.001 |
| Threonine |  | 107.94 | >0.001 |  | 666.61 | >0.001 |  | 30.11 | >0.001 |
| Glutamine |  | 77.724 | >0.001 |  | 82.327 | >0.001 |  | 9.121 | >0.001 |
| Citrate |  | 0.273 | 0.613 |  | 5.245 | 0.613 |  | 0.63 | 0.768 |

| Table S2: Results of 2-way ANOVA testing the effects of sulfide exposure and time on Phytol and MDA levels; Test statistics underlying Fig 4 | | | | | | | | | |
| --- | --- | --- | --- | --- | --- | --- | --- | --- | --- |
| Source of variation |  | Treatment | |  | Time | |  | Treatment x Time | |
|  |  | F | p |  | F | p |  | F | p |
| Phytol |  | 1009 | >0.001 |  | 283.8 | >0.001 |  | 119.3 | >0.001 |
| MDA |  | 309.9 | >0.001 |  | 55.8 | >0.001 |  | 52. | >0.001 |
