# Supplementary figures and images for: Detrimental impact of sulfide on the seagrass Zostera marina in dark hypoxia
Source: PLoS One. 2023 Dec 7;18(12):e0295450. doi: 10.1371/journal.pone.0295450 (PMC10703230; doi:10.1371/journal.pone.0295450)

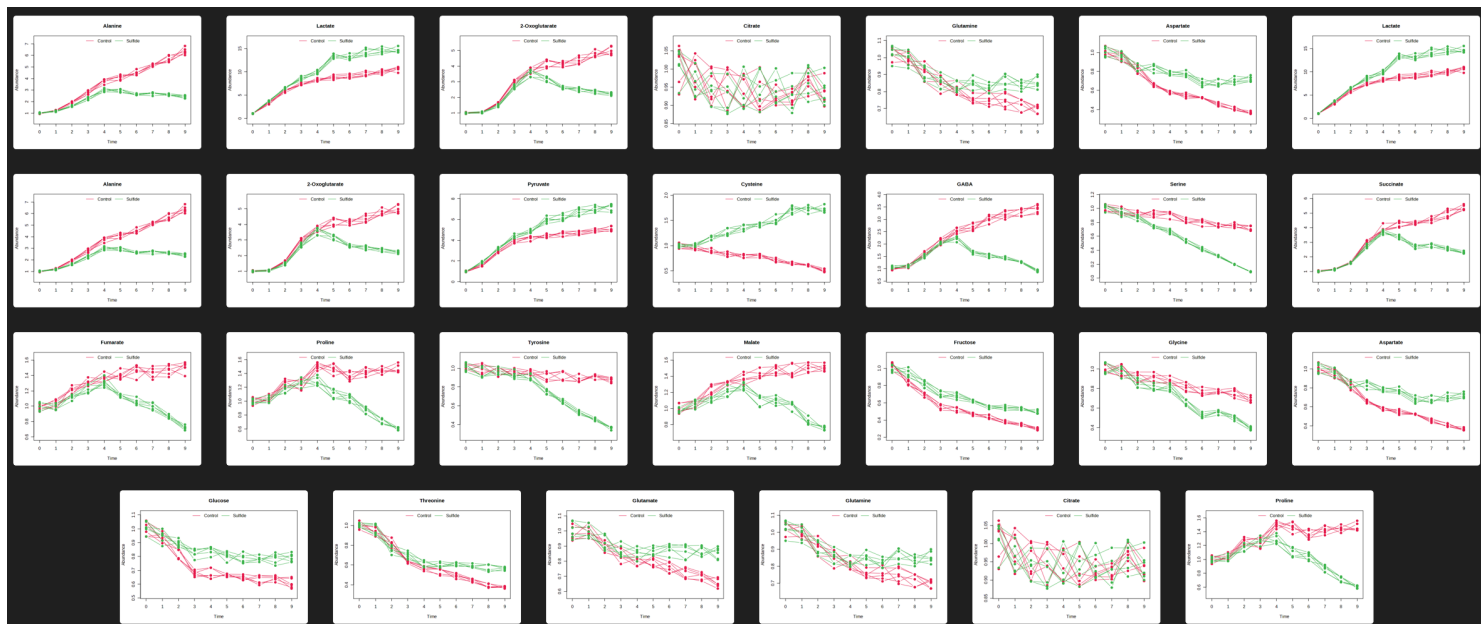

Supplement: S2 File — (PDF) [file pone.0295450.s003.pdf]
