# Supplementary material for: Detrimental impact of sulfide on the seagrass Zostera marina in dark hypoxia
Source: PLoS One. 2023 Dec 7;18(12):e0295450. doi: 10.1371/journal.pone.0295450 (PMC10703230; doi:10.1371/journal.pone.0295450)
Supplement: Attachment — Submitted filename: Response to reviewers.pdf [file pone.0295450.s004.pdf]

Dear Academic Editor Dr. Vasu D. Appanna and Reviewers,

First and foremost, I sincerely apologize for the incomplete data in the submitted manuscript. This oversight occurred due to an error during the final preparation of the documents. Such an oversight should not have happened, and I regret that it was not caught during the proofreading phase. I deeply appreciate your understanding and patience.

Thank you for your constructive feedback on my manuscript titled "Detrimental impact of sulfide on the seagrass Zostera marina in dark hypoxia." I appreciate the time and effort you have dedicated to reviewing my work. I have carefully addressed each comment and made the necessary revisions to improve the clarity and quality of my manuscript. Below, I first provide a general response followed by a point-by-point response to the comments and concerns raised.

Journal Requirements:

1. **Manuscript Style Requirements:**
    - Response: I have revised the manuscript to ensure it aligns with PLOS ONE's style requirements using the provided templates.
2. **Copyediting:**
    - Response: Dr. Lisa Eckfort-Soper, a native speaker and marine ecologist, has copyedited the manuscript with a special focus on clarity and readability.
3. **Methods Section - Permits:**
    - Response: I have provided additional information regarding the permits obtained for the work in the Ethics section. The full name of the authority that approved the field site access has been included, and I have also provided a statement explaining why no permits were required.
4. **Acknowledgments Section:**
    - Response: I have removed the funding-related text from the Acknowledgments section as per the journal's guidelines.
5. **Data Availability Statement:**
    - Response: I have provided the minimal data set underlying the results described in the manuscript as Supporting Information files.
6. **Reference to Figures 3 and 4:**
    - Response: I have ensured that Figures 3 and 4 are appropriately referenced in the text.

Additional Editor Comments:

- Response: I acknowledge the importance of indicating the statistical significance of the experiments performed. I have addressed this in my revised manuscript and have provided the necessary statistical analyses to validate the results in the Supporting Information.

Reviewer #1 Comments:

1. **Study Setting:**
   - Response: I have specified in the abstract, discussion, and conclusion sections that the study was carried out in vitro.
2. **State of Na2S:**
   - Response: I have clarified that the sulfide solutions were made from Na2S nonahydrate, as described in the method of Pedersen and Kristensen (2015).
3. **Sulfide Concentration:**
   - Response: I have provided further details on how the sulfide concentration was measured and clarified that in the sulfide-free chambers, sulfide levels were below the detection limits of the Cline method.
4. **Statistical Significance:**
   - Response: I have added the necessary statistical significance values (e.g., p-values) to compare the control and experimental dataset.
5. **Conclusion:**
   - Response: I have specified in the conclusion that the experiments were conducted in vitro.

Reviewer #2 Comments:

1. **Figure Captions:**
   - Response: I apologize for the oversight and have included the missing figure captions for Figures 3 and 4 in the revised manuscript.
2. **Y-Axis in Figure 3:**
   - Response: I have clarified that the values present fold-change in Figure 3. This was a clerical error, and I have made the necessary corrections.
3. **Error Bars and Statistical Analysis:**
   - Response: I have added error bars to the pathway analysis in Figure 3 for clarity. Additionally, I have provided the underlying data, the 2-way ANOVA test-statistics, and illustrations of all metabolites showing all replicates.
4. **Phytol or MDA Significance:**
   - Response: I have clarified that the changes in phytol and MDA are significant. The necessary statistical analyses have been provided, and I have also included details on how these experiments were performed in the methodology section.

Thank you for your constructive feedback on my manuscript titled "Detrimental impact of sulfide on the seagrass Zostera marina in dark hypoxia." I appreciate the time and effort you have dedicated to reviewing my work. I have carefully addressed each comment and made the necessary revisions to improve the clarity and quality of my manuscript. Below, I provide a point-by-point response to the comments and concerns raised.

Reviewer #1 Comments and Responses:

1. **Comment (Abstract, Discussion, Conclusion sections):** The study was carried out in a seawater aquarium. Therefore, it is important to specify in the abstract, discussion, and conclusion sections that the study was carried out in vitro.
   Response: I acknowledge this oversight and have now explicitly mentioned in the abstract, discussion, and conclusion sections that the study was conducted in vitro.
2. **Comment (Introduction):** Specify the rationale for the selection of Zostera marina along with the morphotype used in this study.
   Response: I appreciate the suggestion. The rationale for selecting Zostera marina L. is its ecological significance and widespread presence in the temperate Northern Hemisphere, especially in Denmark. I had elaborated on this in the introduction, emphasizing its relevance for my study. The specific morphotype from the Danish straits was chosen due to its exposure to high sulfide levels, a consequence of eutrophication in the region.
3. **Comment (Line 100):** Specify the state (solid or liquid) of Na2S that was added.
   Response: I apologize for the oversight. The sulfide solutions were prepared from Na2S nonahydrate in its solid form. I have now clarified this in the methods section.
4. **Comment (Line 101):** Was the chamber 100% free from sulfide? How was the sulfide concentration measured?
   Response: In my experiments, the "sulfide-free" chambers had sulfide levels below the detection limits of the Cline method, which I used for measurement. I have had provided this clarification in the manuscript.
5. **Comment (Lines 150-151):** Provide numerical values for "low micromolar levels."
   Response: I have amended the manuscript to specify the range as 10-100µM.
6. **Comment (Lines 153-154):** Specify the normal physiological sulfide level.
   Response: I have added the observed levels, which were around 325µM.
7. **Comment (Lines 170-172, 172-173, 269-273 & 273-277):** Provide statistical significance values (e.g., p-values) for various datasets.
   Response: I have now included the missing statistical information, including p-values, in the manuscript. Additionally, the supplementary material now contains the raw data and ANOVA test-statistics. In the context of PCA (Fig2), p-values are not typically calculated because the analysis does not involve a probabilistic model that allows for the computation of statistical significance in the way that, say, a t-test or ANOVA would. The focus is rather on the explained variance by each principal component, which is a different concept from statistical significance.
8. **Comment (Conclusion):** Specify in the conclusion that the experiments were conducted in vitro. **Response:** I concur with your suggestion and have made the necessary amendments in the conclusion section.

Reviewer #2 Comments and Responses:

1. **Comment:** There are no figure captions for figure 3 or 4.
   Response: I apologize for this oversight. The missing captions have been added to the revised manuscript.
2. **Comment (Figure 3):** Y-axis is listed as either change or ratio. Why is that?
   Response: The values represent fold-change. I have now clarified this in the figure caption and corrected the clerical error.
3. **Comment (Figure 3):** No error bars for any of the metabolites or statistical analysis.
   Response: For clarity in the pathway analysis (Figure 3), I omitted error bars. However, based on your feedback, I have now provided the underlying data, 2-way ANOVA test-statistics, and illustrations of all metabolites with their replicates in the supplementary material.
4. **Comment:** Are the changes in phytol or MDA significant? What statistical analyses have you done? How were these experiments performed, as I do not see this in the methodology?
   Response: The changes in phytol and MDA were indeed significant. I have now presented the post-hoc test results in the figure and its caption. The raw data and ANOVA test-statistics have also been added to the supplementary material for further clarity.

I believe that the revisions made address the concerns raised by the reviewers and enhance the quality of my manuscript substantially.
I hope that my responses and the changes made to the manuscript will make it suitable for publication in PLOS ONE.

Thank you for considering my work.
Sincerely,
Dr. Hasler-Sheetal
